# Supplementary material for: GATA6 regulates WNT and BMP programs to pattern precardiac mesoderm during the earliest stages of human cardiogenesis
Source: bioRxiv. 2024 Jul 11:2024.07.09.602666. Preprint. [Version 1] doi: 10.1101/2024.07.09.602666 (PMC11257636; doi:10.1101/2024.07.09.602666)
Supplement: Supplementary file 1 [file NIHPP2024.07.09.602666v1-supplement-1.pdf]

bioRxiv preprint doi: https://doi.org/10.1101/2024.07.09.602666; this version posted July 11, 2024. The copyright holder for this preprint (which was not certified by peer review) is the author/funder, who has granted bioRxiv a license to display the preprint in perpetuity. It is made available under aCC-BY 4.0 International license.

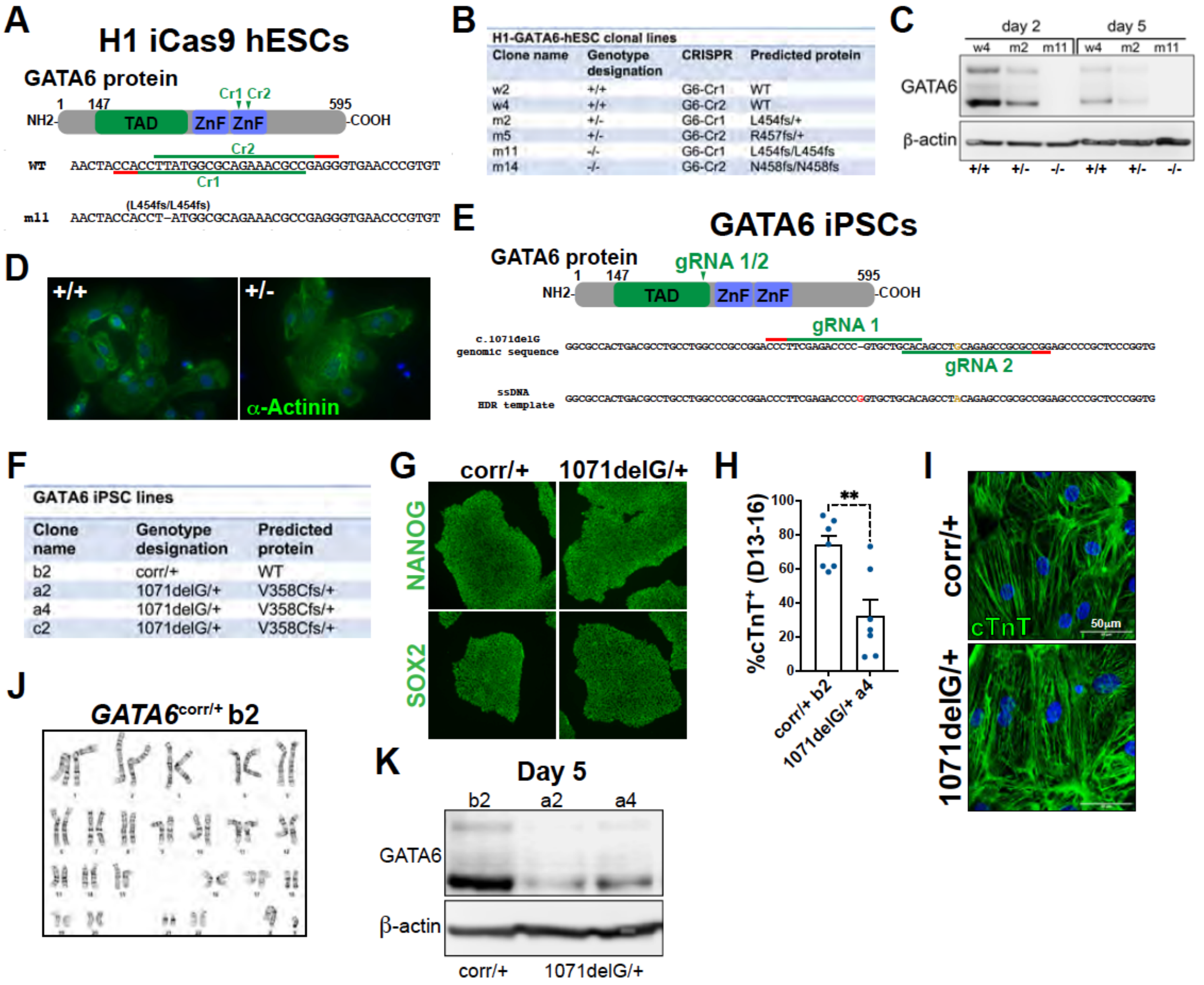

Supplemental Figure 1: CRISPR gene editing and characterization of mutant GATA6 hESC and iPSC lines. (**A**) GATA6 CRISPR targeting scheme using H1 iCas9 hESCs was described in Shi et al. (Ref. 20). Two CRISPR gRNAs were used targeting the C-terminal zinc finger domain (GATA6-Cr1 and Cr2 gRNA target sequence in green, red indicates the PAM). (**B**) Table describing H1-GATA6-hESC clonal lines, genotype designation, and gRNA used in gene editing. Predicted protein describes mutant alleles according to the Human Genome Variation Society (HGVS) guidelines, fs indicates frameshift mutation. (**C**) Western blots from *GATA6*$^{+/+}$, *GATA6*$^{+/-}$, and *GATA6*$^{-/-}$ protein lysates at day 2 or 5 of cardiac differentiation probed for GATA6 with β-actin used as a loading control. (**D**) Immunofluorescence for the sarcomere marker α-Actinin and co-stained with DAPI on day 23 lactate-purified CMs. (**E**) Schematic for CRISPR-based correction of the GATA6 c.1071delG iPSC mutant allele to WT sequence (*GATA6*$^{corr/+}$). A pair of gRNAs flanking the G deletion site were used for targeting with Nickase-Cas9 and a WT repair template to allow for homology directed repair (HDR) of the mutant allele. A single base substitution of G to A (indicated by brown) was used in the WT repair template to induce a silent mutation in the gRNA 2 recognition sequence to prevent additional Cas9 activity after HDR. (**F**) Table describing

bioRxiv preprint doi: https://doi.org/10.1101/2024.07.09.602666; this version posted July 11, 2024. The copyright holder for this preprint (which was not certified by peer review) is the author/funder, who has granted bioRxiv a license to display the preprint in perpetuity. It is made available under aCC-BY 4.0 International license.

the GATA6 iPSC clonal lines. The GATA6 c.1071delG mutant allele is indicated as the protein V358Cfs (according to HGVS guidelines). (**G**) Immunofluorescence for the pluripotency markers NANOG or SOX2 on *GATA6*$^{corr/+}$ or *GATA6*$^{1071delG/+}$ iPSC colonies. (**H**) Flow cytometry quantification for %cTnT^{+} CMs following cardiac directed differentiation for days 13 to 16 of *GATA6*$^{corr/+}$ or *GATA6*$^{1071delG/+}$ iPSCs. Data represents the mean ± SEM, dots represent independent biological replicates. Significance defined as **p<0.01 using the two-tailed Student's T-test. (**I**) Immunofluorescence for cTnT and co-stained with DAPI on day 23 lactate-purified iPSC-CMs. (**J**) Representative karyogram for *GATA6*$^{corr/+}$ iPSCs. (**K**) Western blots from *GATA6*$^{corr/+}$ or *GATA6*$^{1071delG/+}$ protein lysates at day 5 of cardiac differentiation probed for GATA6 with β-actin used as a loading control.

bioRxiv preprint doi: https://doi.org/10.1101/2024.07.09.602666; this version posted July 11, 2024. The copyright holder for this preprint (which was not certified by peer review) is the author/funder, who has granted bioRxiv a license to display the preprint in perpetuity. It is made available under aCC-BY 4.0 International license.

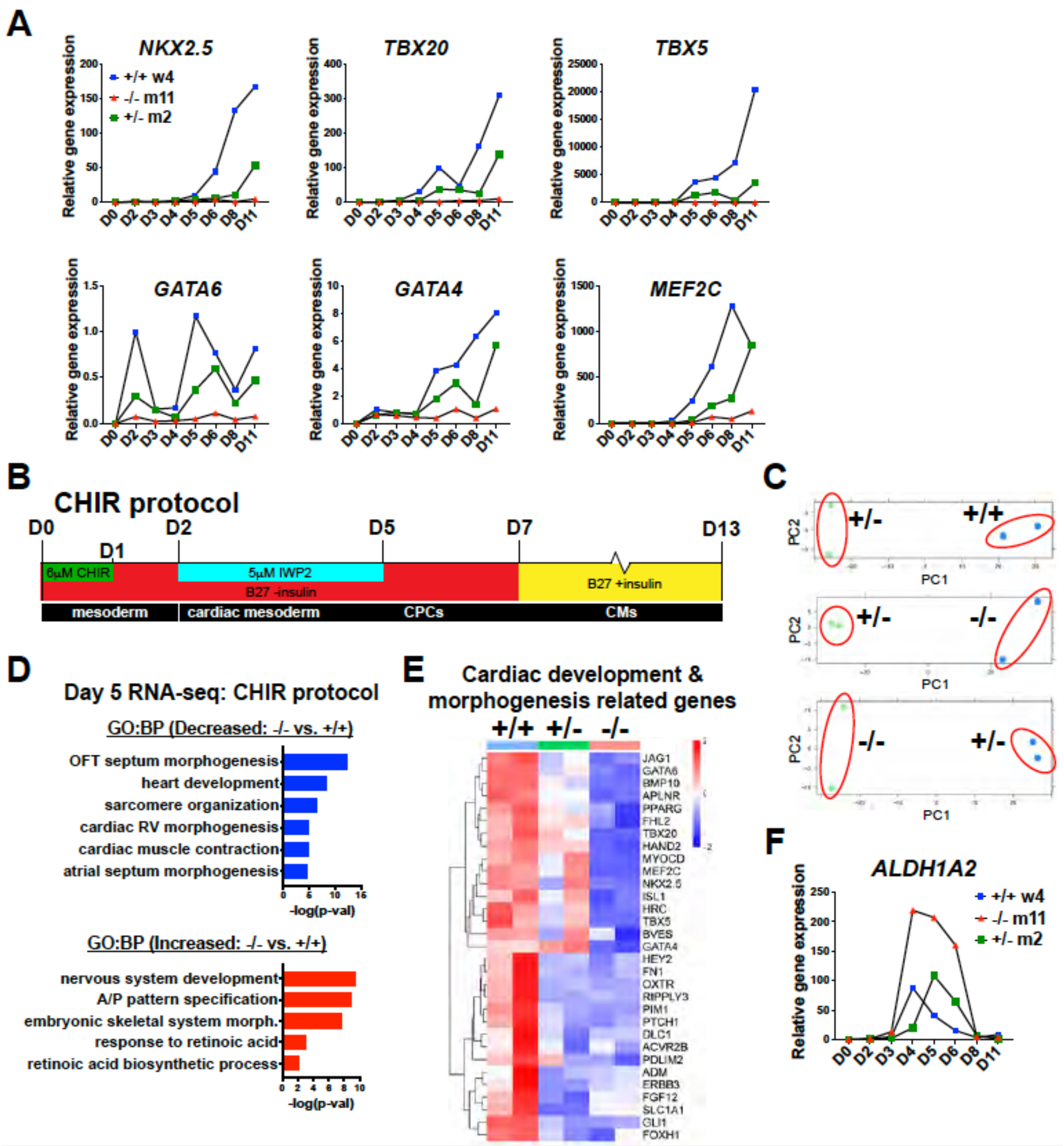

Supplemental Figure 2: CPC marker gene transcriptional analysis of GATA6 WT or mutant hESCs. (**A**) RT-qPCR time course for CPC markers (and *GATA6*) in differentiating *GATA6*$^{+/+}$, *GATA6*$^{+/-}$, and *GATA6*$^{-/-}$ hESCs (normalized to day 2 WT gene expression levels). (**B**) Schematic for *in vitro* CM directed differentiation CHIR protocol. (**C**) Principal component analysis (PCA) for pairwise comparisons of day 5 bulk RNA-seq from *GATA6*$^{+/+}$, *GATA6*$^{+/-}$, and *GATA6*$^{-/-}$ hESCs using the CHIR protocol (n=2). (**D**) Gene ontology (GO) analysis for biological process (BP) using differentially expressed genes (DEGs) from day 5 RNA-seq comparing *GATA6*$^{-/-}$ to WT. (**E**) Heatmap for cardiac development related genes from day 5 RNA-seq. Color gradient indicates relative gene expression level. (**F**) RT-qPCR time course for *ALDH1A2* transcript levels in differentiating *GATA6*$^{+/+}$, *GATA6*$^{+/-}$, and *GATA6*$^{-/-}$ hESCs using the cytokine-based protocol.

bioRxiv preprint doi: https://doi.org/10.1101/2024.07.09.602666; this version posted July 11, 2024. The copyright holder for this preprint (which was not certified by peer review) is the author/funder, who has granted bioRxiv a license to display the preprint in perpetuity. It is made available under aCC-BY 4.0 International license.

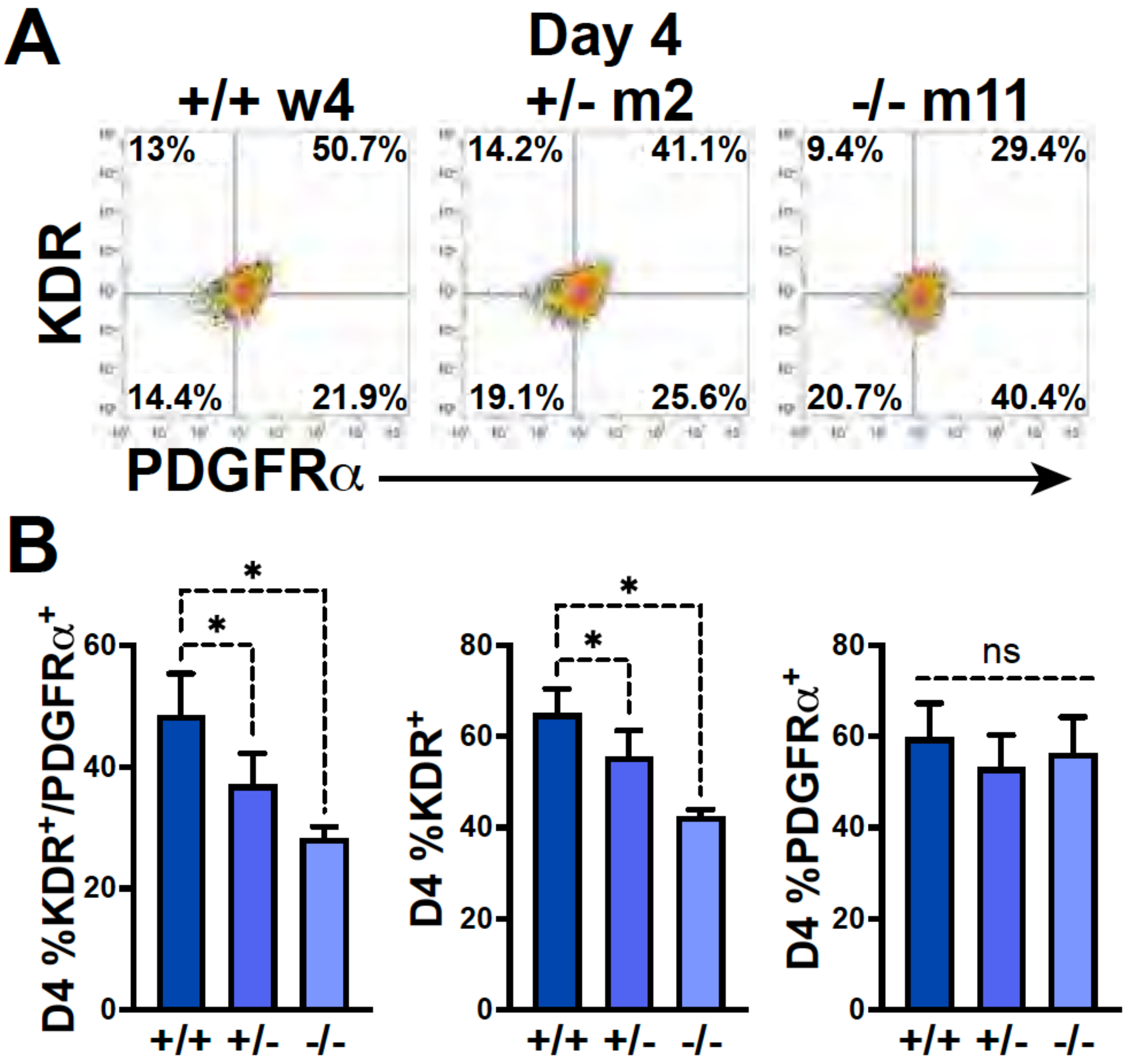

Supplemental Figure 3: KDR and PDGFRα cardiac mesoderm analysis at day 4. (**A**) Representative flow cytometry plots at day 4 of cardiac differentiation analyzing the % KDR and PDGFRα double-positive cells (%K^+P^+) from *GATA6*$^{+/+}$, *GATA6*$^{+/-}$, or *GATA6*$^{-/-}$ hESCs. (**B**) Quantification of day 4 flow cytometry data for %K^+P^+ double-positive, %KDR^+ single positive, or %$PDGFR\alpha^+$ single positive cells (n=6). Data represent the mean ± SEM, with statistical significance indicated as *$p<0.05$ and ns indicating not significant by one-way repeated measures ANOVA with Holm-Šídák's multiple comparison test.

bioRxiv preprint doi: https://doi.org/10.1101/2024.07.09.602666; this version posted July 11, 2024. The copyright holder for this preprint (which was not certified by peer review) is the author/funder, who has granted bioRxiv a license to display the preprint in perpetuity. It is made available under aCC-BY 4.0 International license.

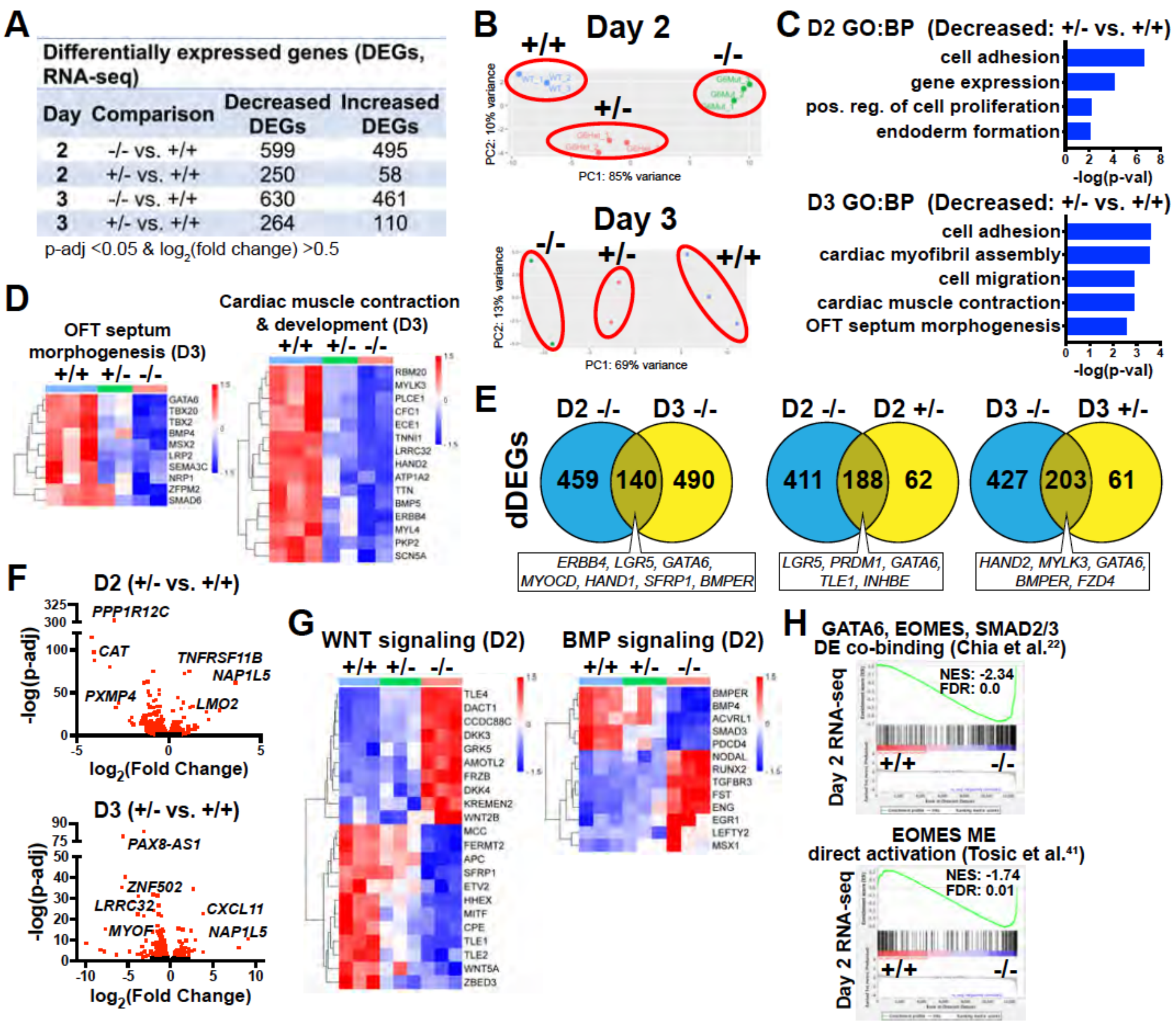

Supplemental Figure 4: RNA-seq analysis following cardiac differentiation at day 2 or day 3. (**A**) Number of DEGs identified when comparing *GATA6*$^{+/-}$ and *GATA6*$^{-/-}$ samples to *GATA6*$^{+/+}$ controls from day 2 and 3 RNA-seq data. **B**) PCA analysis for *GATA6*$^{+/+}$, *GATA6*$^{+/-}$, and *GATA6*$^{-/-}$ profiles from day 2 or day 3 RNA-seq. Data is representative of at least 2 independent biological replicates per sample. (**C**) Day 2 and 3 GO (BP) analysis using dDEGs identified comparing *GATA6*$^{+/-}$ to WT samples. (**D**) Heatmaps for OFT septum morphogenesis and cardiac development related genes from day 3 RNA-seq. Color gradient indicates relative gene expression levels. (**E**) Venn diagram comparisons of dDEGs (relative to WT) identified by day 2 or day 3 RNA-seq of *GATA6*$^{+/-}$ and *GATA6*$^{-/-}$ samples. (**F**) Volcano plots for day 2 or 3 *GATA6*$^{+/-}$ sample RNA-seq gene expression data relative to WT. Dots represent genes, red indicates p-adj<0.05 and black indicates p-adj>0.05. (**G**) Heatmaps for WNT and BMP related genes from day 2 RNA-seq. (**H**) Gene set enrichment analysis (GSEA) analysis of day 2 RNA-seq gene expression data (*GATA6*$^{-/-}$ relative to WT) using the GATA6, EOMES, and SMAD2/3 co-binding during hESC-DE differentiation dataset from Chia et al. (Ref. 22) and EOMES ME direct activation dataset from Tosic et al. (Ref. 41). NES indicates normalized enrichment score; FDR indicates false discovery rate.

bioRxiv preprint doi: https://doi.org/10.1101/2024.07.09.602666; this version posted July 11, 2024. The copyright holder for this preprint (which was not certified by peer review) is the author/funder, who has granted bioRxiv a license to display the preprint in perpetuity. It is made available under aCC-BY 4.0 International license.

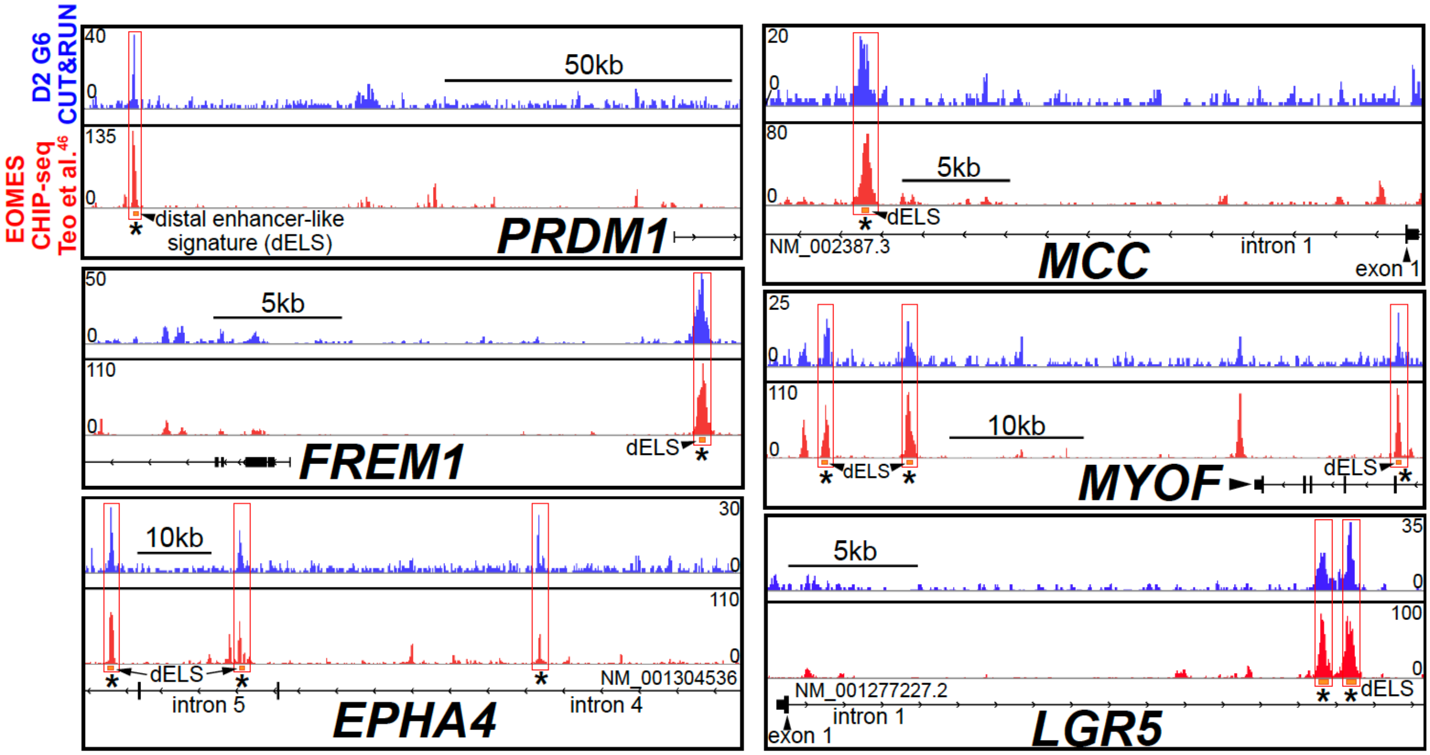

Supplemental Figure 5: GATA6 CUT&RUN peaks overlap with previously published EOMES CHIP-seq peaks. Human genome browser representations of GATA6 CUT&RUN data at day 2 of cardiac differentiation (blue tracks) and EOMES CHIP-seq identified genes at day 2 of hESC-DE differentiation from Teo et al. (Ref. 46) (red tracks). Orange rectangles represent approximate location for distal enhancer-like signatures (dELS) via the ENCODE Project (indicated by black arrows). Asterisks indicate significant GATA6 binding peaks ($p<0.003$ relative to IgG controls) and significant EOMES binding peaks as defined by Teo et al. (Ref. 46).

bioRxiv preprint doi: https://doi.org/10.1101/2024.07.09.602666; this version posted July 11, 2024. The copyright holder for this preprint (which was not certified by peer review) is the author/funder, who has granted bioRxiv a license to display the preprint in perpetuity. It is made available under aCC-BY 4.0 International license.

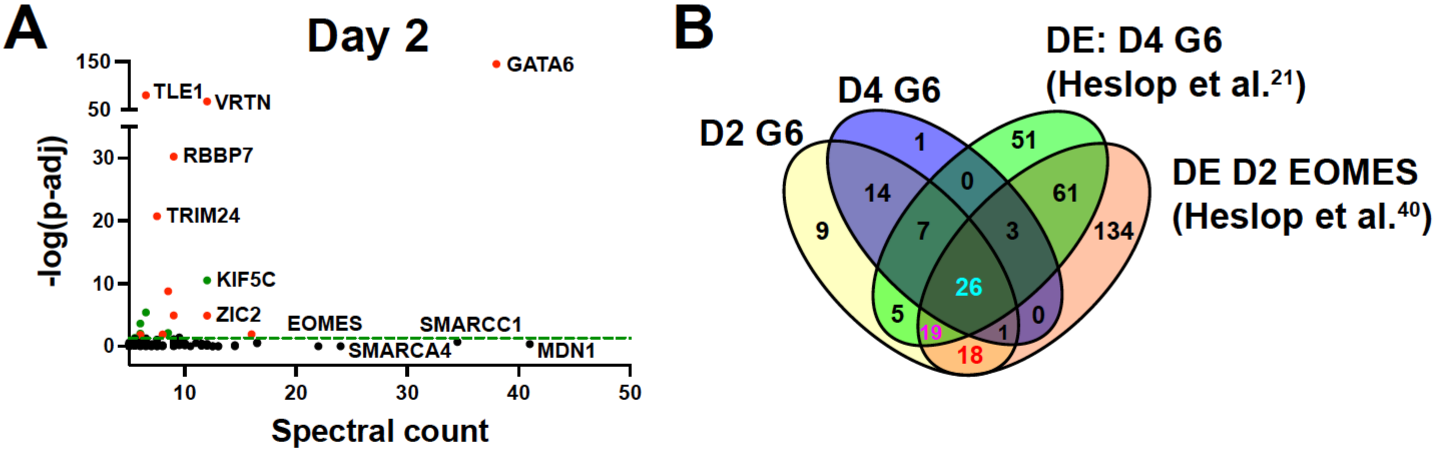

C

Quadruple overlap

GATA6	HDAC2	RBM39
SMARCA4	MTA1	MTA2
GTF2I	CBX1	SMARCD1
FUBP3	CTBP2	TRIM24
MSH6	DDX42	HDAC1
SSRP1	GATAD2A	MBD3
VRTN	MTA3	MSH2
KDM1A	RBBP7	RBM4
SALL4	CTBP1	

D2 G6 & DE: D2 EOMES[40]

ZIC2	CECR2
SMC3	PTBP2
SEPHS1	PSMD5
RCOR2	RECQL
WDHD1	SIN3A
MIXL1	SMARCB1
OGT	ARID1B
SMARCC2	DNAJC8
SART3	RNF40

D2 G6, DE: D4 G6[21], DE: D2 EOMES[40]

MDN1	FEN1
EOMES	SMARCE1
SMC1A	TLE1
NSUN2	TRIM33
XPO5	U2SURP
ACTL6A	PSME3
EP300	SRRT
SMARCD2	HAT1
PPM1G	PRPF40A
BUB3	

Supplemental Figure 6: Extended GATA6-RIME analysis. (**A**) Graph depicts GATA6-RIME enriched proteins (spectral count) identified at day 2 of cardiac differentiation and their corresponding day 2 RNA-seq gene expression level significance (p-adj, *GATA6*$^{-/-}$ relative to WT). Dots indicate genes, black indicates p-adj>0.05, red indicates decreased expression level (p-adj<0.05), green indicates increased expression level (p-adj<0.5). (**B**) Venn diagram comparisons for GATA6-RIME enriched proteins from day 2 (D2 G6) and day 4 (D4 G6) of cardiac differentiation from the present study with day 4 GATA6-RIME (DE: D4 G6) or day 2 EOMES-RIME (DE: D2 EOMES) enriched proteins reported during hESC-DE differentiation from Heslop et al. (Ref. 21 and Ref. 39). (**C**) Proteins identified in the Venn diagram comparisons of RIME data described in (B).

bioRxiv preprint doi: https://doi.org/10.1101/2024.07.09.602666; this version posted July 11, 2024. The copyright holder for this preprint (which was not certified by peer review) is the author/funder, who has granted bioRxiv a license to display the preprint in perpetuity. It is made available under aCC-BY 4.0 International license.

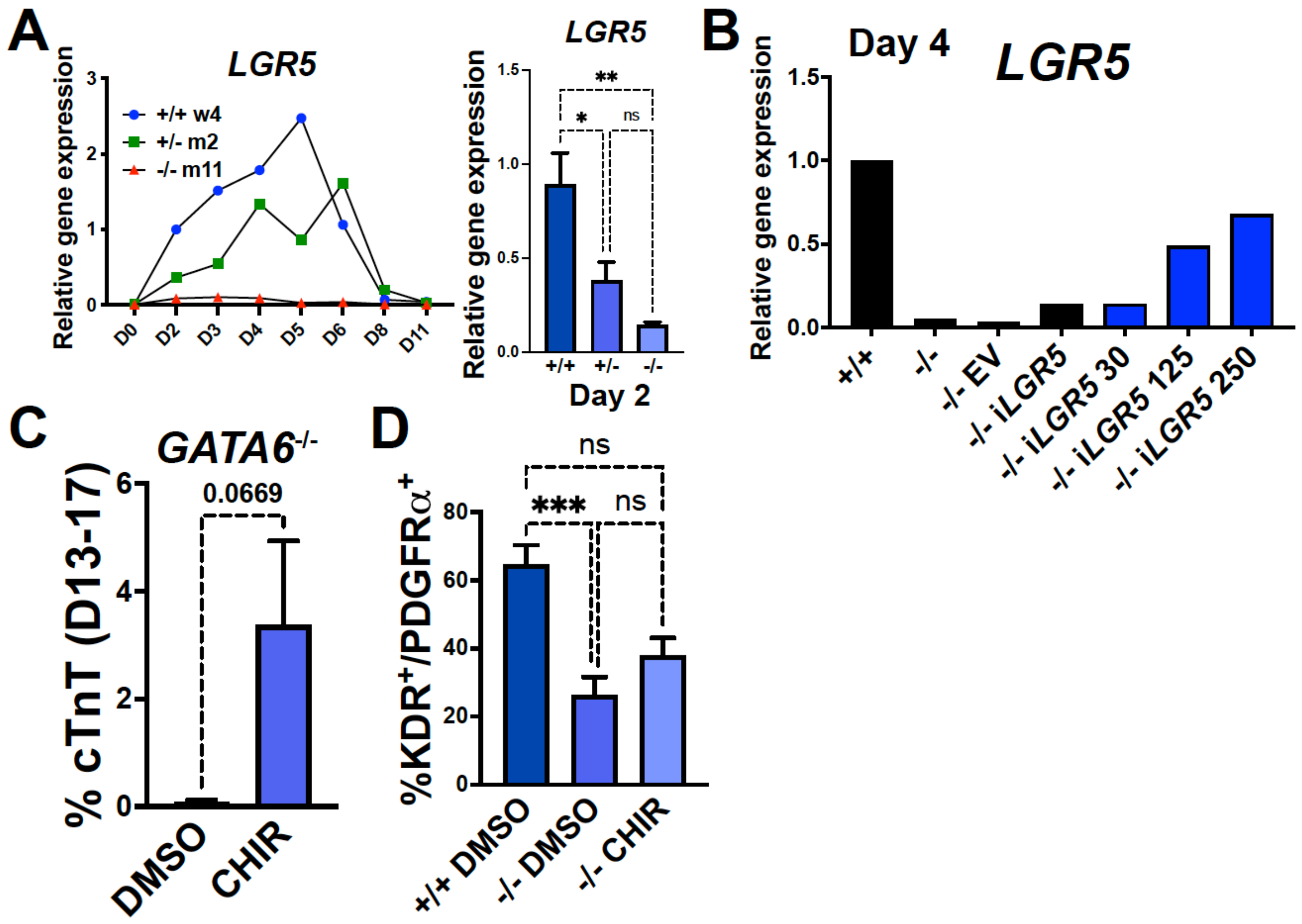

Supplemental Figure 7: Extended data for i*LGR5* and early CHIR treatment. (**A**) RT-qPCR time course for relative *LGR5* expression in differentiating *GATA6*$^{+/+}$, *GATA6*$^{+/-}$, and *GATA6*$^{-/-}$ hESCs normalized to day 2 WT gene expression level (on left). Day 2 RT-qPCR quantification for relative *LGR5* expression level normalized to WT (on right, n=6). (**B**) Day 4 RT-qPCR for relative *LGR5* expression level (normalized to WT) in i*LGR5* or EV transduced *GATA6*$^{-/-}$ hESCs treated with or without varying concentrations of DOX (30, 125, and 250 indicate ng/mL concentrations of DOX). (**C**) %cTnT^{+} CMs from days 13 to 17 of cardiac differentiation quantified by flow cytometry in *GATA6*$^{-/-}$ cells treated with CHIR (3μM) or vehicle (n≥8). (**D**) Flow cytometry quantification for %K^{+}P^{+} double-positive cells at day 5 of cardiac differentiation from *GATA6*$^{-/-}$ hESCs treated with CHIR and vehicle treated *GATA6*$^{+/+}$ or *GATA6*$^{-/-}$ hESCs controls (n≥3). Data represents the mean ± SEM, significance indicated by *p<0.05, **p<0.01, ***p<0.001, by two-tailed Student's t-test (C) or one-way ANOVA with Tukey's multiple comparisons test (A, D).

bioRxiv preprint doi: https://doi.org/10.1101/2024.07.09.602666; this version posted July 11, 2024. The copyright holder for this preprint (which was not certified by peer review) is the author/funder, who has granted bioRxiv a license to display the preprint in perpetuity. It is made available under aCC-BY 4.0 International license.

Supplemental Table 1: GATA6-RIME Enriched Proteins

99 common elements in Day_2_G6_R1 and Day_2_G6_R2		
Gene Name	Protein Name	Spectral Count (Avg)
MDN1	Midasin	41
GATA6	Transcription factor GATA-6	38
SMARCC1	SWI/SNF complex subunit SMARCC1	34.5
SMARCA4	Transcription activator BRG1	24
EOMES	Eomesodermin homolog	22
KLC3	Kinesin light chain 3	16.5
MRPL15	39S ribosomal protein L15 mitochondrial	16.5
DZIP3	E3 ubiquitin-protein ligase DZIP3	16
DNM2	Dynamin-2	14.5
GTF2I	General transcription factor II-I	14.5
FUBP3	Far upstream element-binding protein 3	13
KIF5B	Kinesin-1 heavy chain	13
MSH6	DNA mismatch repair protein Msh6	12.5
SSRP1	FACT complex subunit SSRP1	12.5
ARID1A	AT-rich interactive domain-containing protein 1A	12
GADD45GIP1	Growth arrest and DNA damage-inducible proteins-interacting protein 1	12
KIF5C	Kinesin heavy chain isoform 5C	12
VRTN	Vertnin	12
ZIC2	Zinc finger protein ZIC 2	12
KDM1A	Lysine-specific histone demethylase 1A	11.5
SALL4	Sal-like protein 4	11.5
TOP2B	DNA topoisomerase 2 (Fragment)	11.5
HDAC2	Histone deacetylase 2	11
MRPL44	39S ribosomal protein L44 mitochondrial	10.5
ELAVL1	ELAV-like protein 1	10
MTA1	Metastasis-associated protein MTA1	10
SMC1A	Structural maintenance of chromosomes protein	10
CBX1	Chromobox protein homolog 1	9.5
PYCR2	Pyrroline-5-carboxylate reductase 2	9.5
SMARCA5	SWI/SNF-related matrix-associated actin-dependent regulator of chromatin subfamily A member 5	9.5
SMC3	Structural maintenance of chromosomes protein 3	9.5
CTBP2	C-terminal-binding protein 2	9
DDX42	ATP-dependent RNA helicase DDX42	9
GATAD2A	Transcriptional repressor p66-alpha	9
MTA3	Metastasis-associated protein MTA3	9
NSUN2	tRNA (cytosine(34)-C(5))-methyltransferase	9
RBBP7	Histone-binding protein RBBP7	9
SF1	Splicing factor 1	9
SMU1	WD40 repeat-containing protein SMU1	9
XPO5	Exportin-5	9
ACTL6A	Actin-like protein 6A	8.5
SEPHS1	Selenide water dikinase 1	8.5
CTBP1	C-terminal-binding protein 1	8
EP300	Histone acetyltransferase p300	8
RBM39	RNA-binding protein 39	8
RCOR2	REST corepressor 2	8
SMARCD2	SMARCD2 protein	8
DAZAP1	DAZ-associated protein 1	7.5
HNRNPUL1	Heterogeneous nuclear ribonucleoprotein U-like protein 1	7.5
MRPL4	39S ribosomal protein L4 mitochondrial (Fragment)	7.5
MTA2	Metastasis-associated protein MTA2	7.5
SMARCD1	SWI/SNF-related matrix-associated actin-dependent regulator of chromatin subfamily D member 1	7.5
TRIM24	Transcription intermediary factor 1-alpha	7.5
WDHD1	WD repeat and HMG-box DNA-binding protein 1	7.5
HDAC1	Histone deacetylase 1	7
MIXL1	Homeobox protein MIXL1	7
OGT	UDP-N-acetylglucosamine--peptide N-acetylglucosaminyltransferase 110 kDa subunit	7
PPM1G	Protein phosphatase 1G	7
SMARCC2	SWI/SNF complex subunit SMARCC2	7
XRN2	5'-3' exoribonuclease 2	7
BUB3	Mitotic checkpoint protein BUB3 (Fragment)	6.5
DUT	DUTP pyrophosphatase isoform CRA_c	6.5
FEN1	Flap endonuclease 1	6.5
MBD3	Methyl-CpG-binding domain protein 3	6.5
MSH2	DNA mismatch repair protein Msh2	6.5
RBM4	RNA-binding protein 4	6.5
SART3	Squamous cell carcinoma antigen recognized by T-cells 3	6.5
SMARCE1	SWI/SNF-related matrix-associated actin-dependent regulator of chromatin subfamily E member 1 (Fragment)	6.5
SNX9	Sorting nexin-9	6.5
THRAP3	Thyroid hormone receptor-associated protein 3	6.5
TLE1	Transducin-like enhancer protein 1	6.5
TRIM33	E3 ubiquitin-protein ligase TRIM33 (Fragment)	6.5
U2SURP	U2 snRNP-associated SURP motif-containing protein	6.5
CECR2	Cat eye syndrome critical region protein 2	6
MRPL38	39S ribosomal protein L38 mitochondrial	6
MRPL47	39S ribosomal protein L47 mitochondrial	6
PSME3	Proteasome activator complex subunit 3	6
PTBP2	Polypyrimidine tract-binding protein 2	6
SRRT	Serrate RNA effector molecule homolog	6
USP9X	Probable ubiquitin carboxyl-terminal hydrolase FAF-X	6
DDX46	Probable ATP-dependent RNA helicase DDX46	5.5
HAT1	Histone acetyltransferase type B catalytic subunit	5.5
HINT1	Histidine triad nucleotide-binding protein 1	5.5
MRPL13	39S ribosomal protein L13 mitochondrial	5.5
MRPL20	39S ribosomal protein L20 mitochondrial	5.5
MRPL50	39S ribosomal protein L50 mitochondrial	5.5
MSI1	RNA-binding protein Musashi homolog 1	5.5
PSMD5	26S proteasome non-ATPase regulatory subunit 5	5.5
RECQL	ATP-dependent DNA helicase Q1	5.5
SIN3A	Paired amphipathic helix protein Sin3a	5.5
SMARCB1	SWI/SNF related matrix associated actin dependent regulator of chromatin subfamily b member 1 isoform CRA_c	5.5
SNRNP70	U1 small nuclear ribonucleoprotein 70 kDa	5.5
ARID1B	AT-rich interactive domain-containing protein 1B	5
DNAJC8	DnaJ homolog subfamily C member 8	5
MRPL43	39S ribosomal protein L43 mitochondrial (Fragment)	5
MRPL49	39S ribosomal protein L49 mitochondrial	5
MSI2	RNA-binding protein Musashi homolog 2	5
PRPF40A	Pre-mRNA-processing factor 40 homolog A	5
RNF40	E3 ubiquitin-protein ligase BRE1B	5
RNF40	E3 ubiquitin-protein ligase BRE1B	5

bioRxiv preprint doi: https://doi.org/10.1101/2024.07.09.602666; this version posted July 11, 2024. The copyright holder for this preprint (which was not certified by peer review) is the author/funder, who has granted bioRxiv a license to display the preprint in perpetuity. It is made available under aCC-BY 4.0 International license.

52 common elements in Day_4_G6_R1 and Day_4_G6_R2		
**Gene Name**	**Protein Name**	**Spectral Count (Avg)**
DNM2	Dynamin-2	24.5
GATA6	Transcription factor GATA-6	24
SMARCC1	SWI/SNF complex subunit SMARCC1	24
PYCR2	Pyrroline-5-carboxylate reductase 2	23.5
DZIP3	E3 ubiquitin-protein ligase DZIP3	21.5
SMARCA4	Transcription activator BRG1	18.5
FUBP3	Far upstream element-binding protein 3	15.5
MRPL15	39S ribosomal protein L15 mitochondrial	13
MRPL44	39S ribosomal protein L44 mitochondrial	13
SNX9	Sorting nexin-9	12
GADD45GIP1	Growth arrest and DNA damage-inducible proteins-interacting protein 1	11.5
SSRP1	FACT complex subunit SSRP1	11.5
KIF5B	Kinesin-1 heavy chain	10.5
MSH6	DNA mismatch repair protein Msh6	9.5
SALL4	Sal-like protein 4	9.5
VRTN	Vertnin	9.5
GTF2I	General transcription factor II-I	9
TRIM24	Transcription intermediary factor 1-alpha	9
ELAVL1	ELAV-like protein 1	8.5
KDM1A	Lysine-specific histone demethylase 1A	8.5
SMU1	WD40 repeat-containing protein SMU1	8.5
HDAC2	Histone deacetylase 2	8
MSI2	RNA-binding protein Musashi homolog 2	7.5
MTA1	Metastasis-associated protein MTA1	7.5
RBBP7	Histone-binding protein RBBP7	7.5
SMARCD1	SWI/SNF-related matrix-associated actin-dependent regulator of chromatin subfamily D member 1	7.5
CBX1	Chromobox protein homolog 1	7
CTBP1	C-terminal-binding protein 1	7
DDX42	ATP-dependent RNA helicase DDX42	7
KLC3	Kinesin light chain 3	7
MSH2	DNA mismatch repair protein Msh2	7
MSI1	RNA-binding protein Musashi homolog 1	7
MTA3	Metastasis-associated protein MTA3	7
SF1	Splicing factor 1	7
TAF15	TATA-binding protein-associated factor 2N	7
CTBP2	C-terminal-binding protein 2	6.5
RBM39	RNA-binding protein 39	6.5
SNRPN	Small nuclear ribonucleoprotein-associated protein N (Fragment)	6.5
TIAL1	Nucleolysin TIAR	6.5
GATAD2A	Transcriptional repressor p66-alpha	6
MRPL47	39S ribosomal protein L47 mitochondrial	6
MTA2	Metastasis-associated protein MTA2	6
RBM4	RNA-binding protein 4	6
TOP2B	DNA topoisomerase 2 (Fragment)	6
USP9X	Probable ubiquitin carboxyl-terminal hydrolase FAF-X	6
ARID1A	AT-rich interactive domain-containing protein 1A	5.5
HDAC1	Histone deacetylase 1	5.5
HNRNPUL1	Heterogeneous nuclear ribonucleoprotein U-like protein 1	5.5
MRPL4	39S ribosomal protein L4 mitochondrial (Fragment)	5.5
ADAR	Double-stranded RNA-specific adenosine deaminase	5
MBD3	Methyl-CpG-binding domain protein 3	5
MRPL49	39S ribosomal protein L49 mitochondrial	5

bioRxiv preprint doi: https://doi.org/10.1101/2024.07.09.602666; this version posted July 11, 2024. The copyright holder for this preprint (which was not certified by peer review) is the author/funder, who has granted bioRxiv a license to display the preprint in perpetuity. It is made available under aCC-BY 4.0 International license.

Supplemental Table 2: Cell lines, primer sequences, and antibodies used.

Cell Lines

Name	Source	Notes
H1-GATA6-hESCs	Parent line: WiCell Research Institute, H1 (WA01), NIHhESC-10-0043	Male
GATA6 c.1071delG iPSCs	Yu et al., 2014 (Ref: 15)	Male
293T	ATCC: human embryonic kidney cells, CRL-11268	
CF1 Mouse Embryonic Fibroblasts, irradiated	MTI Global Stem: CF-1 MEF 2M IRR	

gRNAs sequences

Name	Sequence 5' to 3'	Cell line used	Notes
G6-Cr1	GGCGTTTCTGCGCCATAAGG	H1-GATA6-hESCs	
G6-Cr2	TTATGGCGCAGAAACGCCG	H1-GATA6-hESCs	
gRNA 1 - F	caccgCACAGCCTGCAGAGCCGCGC	GATA6 c.1071delG iPSCs	gRNA target sequence capitalized
gRNA 1 - R	aaacGCGCGGCTCTGCAGGCTGTGc	GATA6 c.1071delG iPSCs	gRNA target sequence capitalized
gRNA 2 - F	caccgGTGCAGCACGGGGTCTCGAA	GATA6 c.1071delG iPSCs	gRNA target sequence capitalized
gRNA 2 - R	aaacTTCGAGACCCCGTGCTGCAc	GATA6 c.1071delG iPSCs	gRNA target sequence capitalized

Primer sequences

Name	Sequence 5' to 3'	Application
G6-iPSC-F	GCCCCTACTCGCCCTACG	PCR genotyping
G6-iPSC-R	CACCCGGACGAGAAAGTCCT	PCR genotyping
LGR5-cloning-F	TTCGTACGTACTTCGGGCACCATGGA	Full Length LGR5 PCR (using day 2 cDNA). Includes BsiWI site.
LGR5-cloning-R	GGCAATTGTTAGAGACATGGGACAAATGCC	Full Length LGR5 PCR (using day 2 cDNA). Includes MfeI site.
LGR5-seq1	GAAAGAAATGCTTTGATGGG	LGR5 Sanger sequencing primer 1
LGR5-seq2	ATGCATTTTCCACTTTGCCA	LGR5 Sanger sequencing primer 2
LGR5-seq3	CTGGTGGGAGAATGGGGTTG	LGR5 Sanger sequencing primer 3
CS-TRE-F	ATTTTCGGGTTTATTACAGG	Lentiviral vector Sanger sequencing primer
CS-TRE-R	GGAAAGGAGCTGACAGGT	Lentiviral vector Sanger sequencing primer
HPRT-F	ACCAGTCAACAGGGGACATAA	RT-qPCR
HPRT-R	CTTCGTGGGGTCCTTTTCACC	RT-qPCR
NKX2.5-F	AGCCGAAAAGAAAGAGCTGTGCG	RT-qPCR
NKX2.5-R	GACCTGCGCCTGCGAGAAGAG	RT-qPCR
TBX20-F	GGCGACGGAGAACACAATCAA	RT-qPCR
TBX20-R	CTGGGCACAGGACGACTTC	RT-qPCR
TBX5-F	AGCAGTGACTTCCTACCAGAAC	RT-qPCR
TBX5-R	TGACATTCTGTGCAGCTCCAT	RT-qPCR
GATA6-F	CTGCGGGCTCTACAGCAAG	RT-qPCR
GATA6-R	GTTGGCACAGGACAATCCAAG	RT-qPCR
GATA4-F	AAAGAGGGGATCCAAACCAG	RT-qPCR
GATA4-R	TTGCTGGAGTTGCTGGAAG	RT-qPCR
MEF2C-F	CTGGTGTAACACATCGACCTC	RT-qPCR
MEF2C-R	GATTGCCATACCCGTTCCCT	RT-qPCR
ALDH1A2-F	GGAGTCCCTTTGACCCCACCACT	RT-qPCR
ALDH1A2-R	CCCTTTCGGCCCAGTCCTTTGC	RT-qPCR
LGR5-F	GTTTCCCGCAAGACGTAACT	RT-qPCR
LGR5-R	CAGCGTCTTCACCTCCTACC	RT-qPCR
T-F	ACCCAGTTCATAGCGGTGAC	RT-qPCR
T-R	CCATTGGGAGTACCCAGGTT	RT-qPCR
EOMES-F	CTGCCCACTACAATGTGTTCG	RT-qPCR
EOMES-R	GCGCCTTTGTTATTGGTGAGTTT	RT-qPCR

Antibodies

Antibody	Source	Application
mouse monoclonal α-Actinin	Sigma-Aldrich A7811	immunocytochemistry 1:200
rabbit polyclonal NANOG	Cell Signaling Technology 3850	immunocytochemistry 1:250
rabbit polyclonal SOX2	Invitrogen 48-1400	immunocytochemistry 1:100
mouse monoclonal cardiac troponin T	Invitrogen MA5-12960	immunocytochemistry 1:200, flow cytometry 1:500
rabbit monoclonal GATA6	Cell Signaling Technology 5851	western blotting 1:1000
mouse monoclonal β-actin	Sigma-Aldrich A1978	western blotting 1:50000
mouse monoclonal anti-human KDR PE conjugated	R&D Systems FAB357P-100	flow cytometry 10μL per million cells
mouse monoclonal anti-human PDGFRα PE conjugated	R&D Systems FAB1264A	flow cytometry 10μL per million cells
goat polyclonal anti-human/mouse Brachyury PE-conjugated	R&D Systems IC2085P	flow cytometry 1:40
rabbit polyclonal SMAD2/3	Cell Signaling Technology 3102	western blotting 1:500
rabbit monoclonal Phospho-SMAD2 (Ser465/467)/SMAD3 (Ser423/425) (D27F4)	Cell Signaling Technology 8828	western blotting 1:500
rabbit monoclonal Phospho-SMAD1 (Ser463/465)/ SMAD5 (Ser463/465)/ SMAD9 (Ser465/467) (D5B10)	Cell Signaling Technology 13820	western blotting 1:500
rabbit monoclonal non-phospho (Active) β-Catenin (Ser33/37/Thr41) (D13A1)	Cell Signaling Technology 8814	western blotting 1:1000
rabbit polyclonal β-Catenin	Cell Signaling Technology 9562	western blotting 1:1000
rabbit monoclonal SMARCC1/BAF155 (D7F8S)	Cell Signaling Technology 11956	western blotting 1:1000
rabbit polyclonal TBR2 / Eomes	Abcam ab23345	western blotting 1:500
goat anti-mouse IgG1 Alexa Fluor™ 488	Invitrogen A-21121	immunocytochemistry 1:500
goat anti-rabbit Alexa Fluor™ 488	Invitrogen A-11008	immunocytochemistry 1:1000
goat anti-mouse Alexa Fluor™ 488	Invitrogen A-10667	immunocytochemistry 1:500
goat anti-mouse Alexa Fluor™ 647	Invitrogen A-21236	flow cytometry 1:400
goat anti-rabbit HRP	Bio-Rad 1706515	western blotting 1:2000 - 1:10000
goat anti-mouse HRP	Bio-Rad 1706516	western blotting 1:2000 - 1:10000
mouse monoclonal IgG1 APC-conjugated	R&D Systems IC002A	flow cytometry 10μL per million cells
mouse monoclonal IgG1 PE-conjugated	R&D Systems IC002P	flow cytometry 10μL per million cells
goat polyclonal IgG PE-conjugated	R&D Systems IC108P	flow cytometry 1:40
